# The affecting factors of COVID‐19 vaccine hesitancy in parents of children with cancer: A cross‐sectional Jordanian study

**DOI:** 10.1002/iid3.1344

**Published:** 2024-07-26

**Authors:** Sawsan Mubarak, Hadeel AlGhawire, Sumaiah AlNaimat

**Affiliations:** ^1^ Infection Control Program King Hussein Cancer Center Amman Jordan; ^2^ Office of Scientific Affairs and Research King Hussein Cancer Center Amman Jordan

**Keywords:** cancer, children, COVID‐19 vaccine, pediatric oncology, vaccine hesitancy

## Abstract

**Background and Objective:**

Children with cancer have higher mortality and morbidity rates than have been reported in general children patients infected by coronavirus infection 2019 (COVID‐19). Thus, for children with cancer, COVID‐19 vaccination is a priority. This study aims to investigate the factors influencing COVID‐19 vaccine hesitancy in parents of children with cancer in Jordan.

**Methods:**

A cross‐sectional study was conducted during the third quarter of 2022 at the King Hussein Cancer Center in Amman, Jordan. The study employed a self‐administered questionnaire, incorporating COVID‐specific questions. Participants included parents of children aged 18 years or younger undergoing treatment or monitoring at the center. A straightforward random sampling approach was used to recruit participants. Ethical approval and institutional permission were obtained, ensuring voluntary participation with the right to withdraw.

**Results:**

A total of 409 participants, predominantly female, were enrolled in the study. Notably, most of the enrolled parents did not intend to have their children vaccinated either for seasonal flu or for COVID‐19, 76.2% and 78.7%, respectively. The bulk of the parents were encouraged to vaccinate their child by the child's pediatrician (82.9%). Parents' age and their children's age were significantly influenced their willingness to vaccinate their children with the COVID‐19 vaccine (*p *< .001), in which parents' age group 45−54 years and children's age group above 15 years old show the highest vaccination rate. Meanwhile, there was a significant association between children's vaccination with parents suffering from chronic disease (*p *< .001) and parents receiving the COVID‐19 vaccine (*p* = .014). There are still some concerns regarding the COVID‐19 vaccine's effectiveness, safety, and whether it is essential for their child.

**Conclusion:**

A large proportion of parents in Jordan are hesitant about the COVID‐19 vaccine when considering its administration to their children with cancer. This finding emphasizes the importance of communication and education to address vaccination hesitancy.

## Graphical abstract

The study investigates coronavirus infection 2019 (COVID‐19) vaccine hesitancy in parents of pediatric cancer patients in Jordan. A total of 409 parents were surveyed through a web‐based questionnaire. Factors such as parents' age and pediatric age were found to play a significant role in their willingness to vaccinate their children with cancer against COVID‐19. A large proportion of parents expressed hesitancy due to concerns about vaccine effectiveness, safety, and importance for their children. The study emphasizes the importance of addressing vaccine hesitancy to improve pediatric vaccination rates and achieve collective immunity.

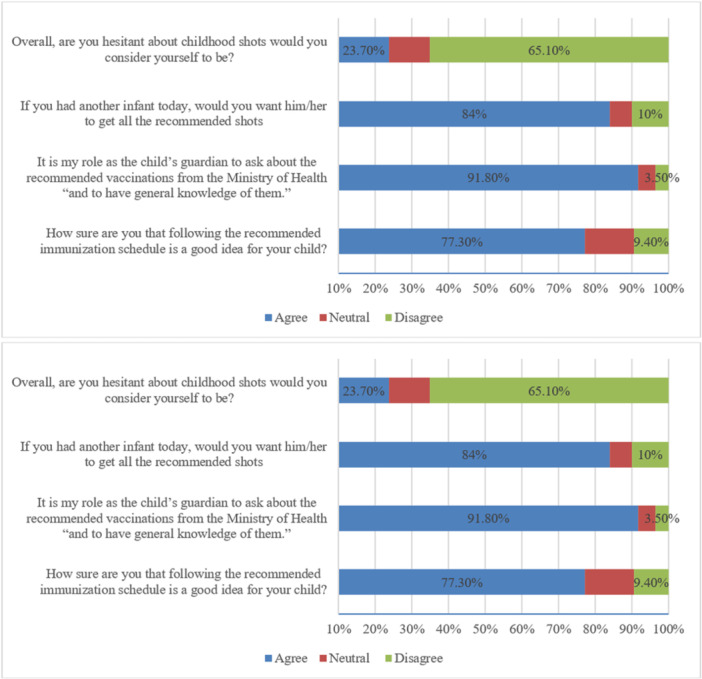

## 1 BACKGROUND/RATIONALE

A cancer diagnosis is upsetting at any age, but especially so when the patient is a child. In the United States in 2022, an estimated 10,470 new cases of cancer were diagnosed among children and adolescents, and about 1050 pediatric patients are expected to die from the disease. The most common types of cancer diagnosed in children and adolescents are leukemia, brain, and other central nervous system tumors, and lymphomas.^1^ The US Department of Health and Human Services and the US Food and Drug Administration reference approximate age ranges for pediatric patients, which consist of the following: infancy: between birth and 2 years of age; childhood: from 2 to 12 years of age; and adolescence: from 12 to 21 years of age.^2^

In December 2019, the new coronavirus infection 2019 (COVID‐19) pandemic made its appearance in the Chinese city of Wuhan. The World Health Organization declared the epidemic a pandemic in March 2020.^3^, ^4^ According to the American Academy of Pediatrics, the overall rate of COVID‐19 pediatric infection in the United States is 19,633 cases per 100,000 children and adolescents, which represents 18.4% of all COVID‐19 cases.^5^ An estimates of US pediatric hospitalizations are 15.8 per 100,000 in infants and children 0–4 years of age and 9.2 per 100,000 in those 5–17 years of age, although the COVID‐19 hospitalization burden is smaller in children than in adults.^6^

Cancer patients are more likely to have severe COVID‐19, according to various studies. Cancer patients are more susceptible to COVID‐19 infection due to immunosuppression caused by several therapeutic procedures such as chemotherapy, radiation, and stem cell transplantation. For example, a retrospective cohort study found that cancer patients had a higher 30‐day all‐cause mortality rate, which was connected to cancer‐specific risk factors.^7^ In another study of patients with cancer and COVID‐19 infection, patients with tumors showed an increased risk of developing severe COVID‐19 infection, poor clinical outcomes, and death.^8^ Along the same lines, the immune systems of children with cancer are affected by the tumor and antineoplastic medications, that may lead to chemotherapeutic regimen changes or even deferral of it as a consequence of COVID‐19.^9^ Such evidence makes cancer patients a priority for COVID‐19 vaccination.

Broadly speaking, concerning children patients, the COVID‐19 clinical status is usually mild; nevertheless, severe cases may occur, with a reported frequency of 1%–8%, and 25% of severe cases in children and adolescents may require ICU admission. It has been found that patients younger than 1 year of age and children with comorbidities are at a higher risk of developing a severe illness and being admitted to the ICU.^10^, ^11^ A systematic review of COVID‐19 in the pediatric oncology setting, in particular, found that around 10% of children and adolescents required critical care, and 32% of them needed oxygen. Moreover, 4.9% of youngsters lost their lives as a result of COVID‐19.^9^ In the same manner, it was observed that pediatric cancer patients infected by COVID‐19 have higher severity, mortality, and morbidity rates than what was reported in general pediatric COVID‐19 cases.^9^, ^12^ To control COVID‐19, this information can aid in risk categorization.^9^ Thus, children and adolescents with underlying medical conditions or chronic diseases are at increased risk for severe illness, so public health prevention and vaccine prioritization efforts might consider the potential for severe COVID‐19 illness among children and adolescents with these underlying medical conditions and chronic diseases.^13^ A COVID‐19 vaccine campaign targeting pediatric cancer patients could dramatically reduce hospitalization and racial disparities from COVID‐19.^12^ The current efficacious vaccine is an essential tool in the fight against the current COVID‐19 pandemic to achieve collective immunity, which can help reduce transmission, hospitalizations, and intensive care utilization, as well as prevent additional mortality.^14^ A study assessed the impact of COVID‐19 vaccination in Italy and found that 17% of expected cases, 32% of hospitalizations, 29% of ICU admissions, and 38% of deaths were prevented by vaccination.^15^

Presently, several vaccines are available worldwide with different administration schedules, and efficacies. However, the US Centers for Disease Control and Prevention (CDC) recommends COVID‐19 vaccines for everyone ages 6 months and older. CDC also recommends COVID‐19 vaccines available for children and adolescents, including Pfizer‐BioNTech COVID‐19 vaccines and Moderna COVID‐19 vaccines.^16^

Vaccination has long been recognized as one of the most important public health strategies for preventing viral disease, safeguarding vulnerable populations and the general public, and ultimately saving lives.^17^, ^18^ Among the issues that have been identified to affect vaccine coverage is vaccine hesitancy, which could represent a major challenge in the global efforts to control the pandemic.^19^ Vaccine hesitancy refers to a delay in accepting or refusing vaccination despite its availability.^20^

However, it is commonly acknowledged that, for a variety of reasons, different communities have varying levels of concern about receiving vaccines. Many factors influence vaccination decisions, including trust in the government and healthcare professionals, social influences, high levels of knowledge about the vaccine, and generally positive attitudes toward vaccines.^21^

Although the majority of children and adolescents appear to be unaffected by the disease, many other factors must be considered, including high‐risk subpopulations, the true attributable risk for severe COVID‐19 disease (e.g., co‐infection), and the role that children play in community‐based viral transmission.^22^ Immunization of children and adolescents aims to limit the circulation of the virus by increasing the number of immune individuals, preventing secondary infections in the household, and gradually restoring school attendance and other social activities in this age group.^23^

Many studies around the world have reported hesitancy toward the COVID‐19 vaccine among parents toward pediatric vaccination, including in the United Kingdom, Italy, the United States, and Poland.^23^, ^24^, ^25^, ^26^ Variable levels of vaccine acceptance for COVID‐19 childhood immunization have been observed in China, where the prevalence of parents' willingness to vaccinate their children under the age of 18 years ranges between 72.6% and 44.5%.^27^, ^28^

The success of any COVID‐19 vaccination program will depend on the public willingness to receive the vaccination. For children and adolescents under the age of 18 years who have cancer, parents are usually the decision‐makers regarding their child's vaccination. Hence, it is important to understand parents' willingness to accept children's COVID‐19 vaccination. To our knowledge, there has been no study conducted in Jordan concerning the intention of parents toward their child with cancer vaccination against COVID‐19 infection.

## 2 OBJECTIVE

This study aimed to investigate the affecting factors of COVID‐19 vaccine hesitancy in parents of children with cancer and their attitude toward vaccinations in Jordan; in addition, we investigated the characteristics related to parent‐perceived COVID‐19 vaccine decision‐making and compared them to parent‐perceived vaccine decision‐making for influenza vaccine.

## 3 METHODS

### 3.1 Study design and settings

A cross‐sectional survey was employed in this study to assess COVID‐19 vaccine hesitancy in parents of children and adolescents with cancer during the third quarter of 2022. The study was conducted at King Hussein Cancer Center (KHCC) in Amman, Jordan. A self‐administered questionnaire was used for data collection. For this study, COVID‐specific questions were adopted from the Parent Attitudes about Childhood Vaccines (PACV survey).^29^

A pilot study was conducted before the main study to assess the feasibility and refine the survey instrument. This pilot study involved a small sample of participants who were representative of the target population. The feedback and insights gained from the pilot study were instrumental in finalizing the questionnaire and ensuring its clarity, relevance, and appropriateness for the intended research objectives.

### 3.2 Participants

The study's population targeted all parents of children with cancer aged 18 years or less who are receiving treatment or being monitored in KHCC. The parents of children patients who were actively on cancer medication in our oncology center were contacted during their visits to the center, where we provided them with detailed information about the study and sought their participation.

As for the survey access and completion process, we provided multiple options to accommodate various preferences and circumstances. Parents complete the survey via a traditional paper form, which could be submitted during their subsequent visits to the center.

Regarding the patients included in the study, we specifically targeted children who were actively undergoing cancer treatment at the time of the survey administration. This ensured that our data accurately reflected the experiences and perspectives of families currently grappling with the challenges associated with cancer treatment. Consent was obtained from the participants to participate voluntarily in the study. As well, they had the right to choose not to participate or withdraw from the research survey at any time. Participants' responses were confidential.

Eligible respondents were informed about the aim and significance of the research to encourage their voluntary participation through a cover page. Their acceptance as responders was determined by their engagement and completion of the questionnaire. Consequently, a waiver of documentation was sought, considering the low‐risk nature of the study.

In adherence to ethical standards, participants were duly informed about the utilization and anonymization of their data before their engagement in the study. Additionally, participants were clarified that no identifiers such as names, medical record numbers, or phone numbers were required in the questionnaire, further ensuring the anonymity of their responses and enhancing participant confidentiality.

## 4 VARIABLES/DATA SOURCES

Data were gathered using a straightforward random sampling approach. The questionnaire consists of four sections. The first section was demographic data such as gender, age, education level, nature of the job, health status, and taking the COVID‐19 vaccine. The second section was regarding the practice of all vaccines. The third and fourth sections were items regarding knowledge and attitude toward the COVID‐19 vaccine. Item scores were summed in an unweight fashion to obtain a total raw score. This research was prepared based on the Strengthening the Reporting of Observational Studies in Epidemiology (STROBE) checklist for a cross‐sectional study.

## 5 BIAS

Potential sources of bias include but are not limited to self‐reporting bias in survey responses, selection bias in the sampling method, and potential biases in parental attitudes toward vaccination. The study team has made concerted efforts to address and mitigate these biases where possible, and readers are encouraged to interpret the results with a nuanced understanding of these potential limitations.

## 6 STUDY SIZE

The study population comprised all parents of patients who received treatment or were under monitoring. Utilizing a convenience sampling technique, the study targeted a total of 2773 pediatric patients. The sample size estimation for this study was determined based on a Power of 0.8, a *⍺* level of 0.05, and a medium effect size of 0.5, resulting in a required sample size of 319 participants. To account for a 20% dropout rate due to technical issues, the sample was increased accordingly, leading to a total of 333 participants deemed necessary for the study. Ultimately, it was determined that a study sample of 330 participants was appropriate. Notably, participants' children were not required to have received vaccination before enrollment. Following the completion of the study, the sample size was found to be 409 participants whose demographic information has also been reported, exceeding the initially estimated sample size. We set the response range of several items to ensure the survey's quality (e.g., age, educational level, and so on).

### 6.1 Statistical analysis

The Statistical Package for Social Sciences (IBM‐SPSS) was used to analyze the data. Categorical variables were reported as frequency counts and percentages. Whereas continuous variables were reported as mean and standard deviation. Also, a cross‐tabulation analysis using the chi‐square test was employed to examine significant differences between categorical variables. A *t*‐test and logistic regression analysis were used to examine significant differences between variables. For all analyses, two‐tailed tests were used, and a *p*‐value equal to or less than .05 was considered statistically significant.

## 7 RESULTS

**Figure 1:**
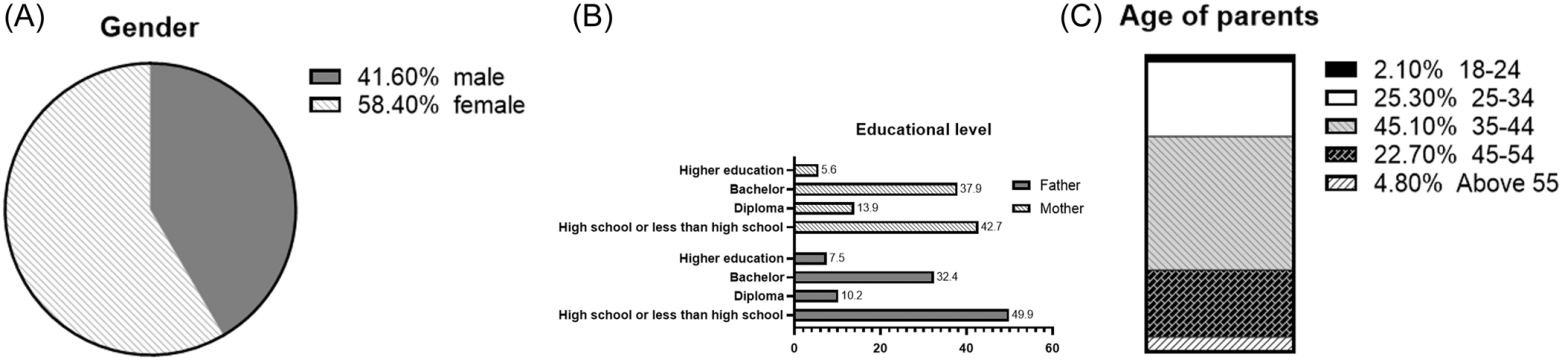
(A) Participants' male/female distribution, (B) participants' educational level, and (C) participants' age.

In this study, a total of 409 participants were recruited, most of them female, as shown in Figure 1A. They had several educational levels, and variable ages as illustrated in Figure 1B,C respectively. It was determined that the preponderance of the participants (89.5%) are not working in the medical field. In addition, only 11.8% of the participants have chronic diseases.

Nearly 62.1% of participants did not receive the influenza vaccine in the last year, while 84.8% of them had no intention to receive it. Whereas the majority 90.2% have received the COVID‐19 vaccine, and 17.5% of those who didn't receive the vaccine declared a definite willingness to receive it in the future. Regarding COVID‐19 infection; 54.3% of the participants got infected with COVID‐19, 45.5% of them (participants who tested positive for COVID‐19 within the entire cohort of participants) had moderate symptoms, and 15.9% of them (entire cohort of participants in our study) lost a family member due to COVID‐19 infection. Most of the children with cancer included in this study were in the 4–7 years age group. Only 3.4% of the children patients have a chronic disease (such as diabetes, asthma, hypertension, and others, excluding cancer from this category), and 42.9% got infected with COVID‐19. However, 83.5% of them received all the prescribed vaccinations according to the national vaccination program. The majority (71.1%) did not receive the flu vaccine, and 89.4% of them didn't receive the COVID‐19 vaccine. Most of the recruited parents did not intend to have their child vaccinated for either seasonal flu or COVID‐19, 76.2% and 78.7%, respectively. The bulk of the parents were encouraged to vaccinate their child by the child's pediatrician (82.9%), as shown in Table 1.

**Table 1:** Respondents' perceptions and practices relating to the COVID‐19 infection and Flu vaccine and COVID‐19 vaccine.

		Frequency	Percentage
Did you receive the flu vaccine in the last year	Yes	155	37.9
	No	254	62.1
If no	I have the intention to get the vaccine	34	15.2
	I don't have the intention to get the vaccine	189	84.8
Did you receive COVID vaccine	Yes	367	90.2
	No	40	9.8
If no	I have the intention to get the vaccine	7	17.5
	I don't have the intention to get the vaccine	33	82.5
Infected with COVID	Yes	222	54.3
	No	187	45.7
Symptoms severity	Mild	67	30.2
	Moderate	101	45.5
	Severe	54	24.3
Family loss due to COVID	Yes	65	15.9
	No	344	84.1
Child's age	0–3	68	16.9
	4–7	125	31
	8–11	79	19.6
	12–15	74	18.4
	Above 15	57	14.1
The child has a chronic disease	Yes	14	3.4
	No	393	96.6
The child received the flu vaccine	Yes	118	28.9
	No	290	71.1
If no	I have the intention to vaccinate my child	62	23.8
	I don't have the intention to vaccinate my child	198	76.2
The child got COVID	Yes	174	42.9
	No	232	57.1
Did your child receive all the prescribed vaccinations according to the national vaccination program?	Yes	338	83.5
	No	67	16.5
The child received COVID vaccine	Yes	43	10.6
	No	362	89.4
If no	I have the intention to vaccinate my child	71	21.3
	I don't have the intention to vaccinate my child	262	78.7
Your decision depends on	The advice of the pediatrician	380	82.9
	Opinions of older people and relatives	9	1.9
	Research results and scientific references	56	12.2
	What is published in the media and social media	13	2.8

Abbreviation: COVID‐19, coronavirus infection 2019.

The association between parents' demographic data and children with cancer who received COVID‐19 vaccination was described in Table 2. There was a significant association between the age of parents and children with cancer who received COVID‐19 vaccination, along with the child's age (*p *< .001), in which parents belonging to the 45–54 years age group and children's age group above 15 years old show the highest vaccination rate. Meanwhile, there was a significant association between children's vaccination with parents suffering from chronic disease (*p *< .001) and parents receiving the COVID‐19 vaccine (*p* = .014).

**Table 2:** Factors associated with children's COVID‐19 vaccination.

		Received COVID vaccine (*n* = 43)		Did not receive COVID vaccine (*n* = 362)		*p* Value
		Frequency	Percentage	Frequency	Percentage	
Parent's gender	Male	21	48.8	146	40.3	.284
	Female	22	51.2	216	59.7	
	Total	43	100	362	100	
Educational level: Father	High school or less than high school	22	51.2	178	50.3	.234
	Diploma	8	18.6	33	9.3	
	Bachelor	11	25.6	117	33.1	
	Higher education	2	4.7	26	7.35	
	Total	43	100	354	100	
Educational level: Mother	High school or less than high school	19	45.2	150	42.7	.363
	Diploma	9	21.4	45	12.8	
	Bachelor	12	28.6	137	39	
	Higher education	2	4.8	19	5.4	
	Total	42	100	351	100	
Age of parents	18−24	0	0	8	2.4	<.001
	25−34	6	15.45	89	26.8	
	35−44	8	20.5	159	47.9	
	45−54	19	48.7	65	19.6	
	Above 55	6	15.4	11	3.3	
	Total	39	100	332	100	
Do you work in the medical field?	Yes	5	11.6	38	10.5	.825
	No	38	88.4	323	89.5	
	Total	43	100	361	100	
Parents suffer from chronic diseases?	Yes	13	30.2	35	9.7	<.001
	No	30	69.8	326	90.3	
	Total	43	100	361	100	
Did you receive the flu vaccine in the last year?	Yes	15	34.9	140	38.7	.741
	No	28	65.1	222	61.3	
	Total	43	100	362	100	
Parent received a COVID‐19 vaccine	Yes	43	100	320	88.9	.014
	No	0	0	40	11.1	
	Total	43	100	360	100	
Parents infected with COVID‐19	Yes	22	51.2	198	54.7	.660
	No	21	48.8	164	45.3	
	Total	43	100	362	100	
Symptoms severity	Mild	5	22.7	61	30.8	.635
	Moderate	12	54.5	88	44.4	
	Severe	5	22.7	49	24.7	
	Total	22	100	198	100	
Family loss due to COVID	Yes	7	16.3	57	15.7	.928
	No	36	83.7	305	84.3	
	Total	43	100	362	100	
Child's age	0−3	1	2.3	66	18.5	<.001
	4−7	5	11.6	119	33.4	
	8−11	7	16.3	72	20.2	
	12−15	9	20.9	63	17.7	
	Above 15	21	48.8	36	10.1	
	Total	43	100	356	100	
The child has a chronic disease	Yes	2	4.7	12	3.3	.656
	No	41	95.3	348	96.7	
	Total	43	100	360	100	
The child received the flu vaccine	Yes	16	37.2	101	28	.207
	No	27	62.8	260	72	
	Total	43	100	361	100	
If no	I have the intention to vaccinate my child	8	33.3	54	23	.314
	I don't have the intention to vaccinate my child	16	66.7	181	77	
	Total	24	100	235	100	
The child gets COVID	Yes	20	46.5	152	42.2	.591
	No	23	53.5	208	57.8	
	Total	43	100	360	100	
Did your child receive all the prescribed vaccinations according to the national vaccination program	Yes	41	95.3	295	82.2	.028
	No	2	4.7	64	17.8	
	Total	43	100	359	100	

Abbreviation: COVID‐19, coronavirus infection 2019.

Table 3 presents factors associated with parental willingness to vaccinate their child who had cancer with the COVID‐19 vaccine. Analysis reveals that parental intention to vaccinate their children was significantly associated with parents' intentions to vaccinate their children with flu vaccine (*p* < .001).

**Table 3:** Factors associated with the willingness of parents to vaccinate their child who had cancer with the COVID‐19 vaccine.

		I have the intention to vaccinate my child with the COVID‐19 vaccine (*n* = 71)		I don't have the intention to vaccinate my child with the COVID‐19 vaccine (*n* = 262)		*p* Value
		Frequency	Percentage	Frequency	Percentage	
Parent's gender	Male	35	49.3	101	38.5	.102
	Female	36	50.7	161	61.5	
	Total	71	100	262	100	
Educational level: Father	High school or less than high school	39	55.7	125	48.6	.251
	Diploma	4	5.7	25	9.7	
	Bachelor	25	35.7	85	33.1	
	Higher education	2	2.9	22	8.6	
	Total	70	100	257	100	
Educational level: Mother	High school or less than high school	28	41.2	111	43	.565
	Diploma	7	10.3	34	13.2	
	Bachelor	31	45.6	98	38	
	Higher education	2	2.9	15	5.8	
	Total	68	100	258	100	
Guardian	18−24	2	2.9	6	2.5	.937
	25−34	20	29.4	63	26.4	
	35−44	32	47.1	114	47.7	
	45−54	11	16.2	48	20.1	
	Above 55	3	4.4	8	3.3	
	Total	68	100	239	100	
Your work in the medical field	Yes	10	14.1	25	9.6	.273
	No	61	85.9	236	90.4	
	Total	71	100	261	100	
Do you suffer from chronic diseases	Yes	7	9.9	23	8.8	.785
	No	64	90.1	238	91.2	
	Total	71	100	261	100	
Did you receive the flu vaccine in the last year	Yes	32	45.1	93	35.5	.139
	No	39	54.9	169	64.5	
	Total	71	100	262	100	
Did you receive COVID vaccine	Yes	65	91.5	228	87.4	.409
	No	6	8.5	33	12.6	
	Total	71	100	261	100	
If no	I have the intention to get the vaccine	2	33.3	5	15.2	.286
	I don't have the intention to get the vaccine	4	66.7	28	84.8	
	Total	6	100	33	100	
Infected with COVID	Yes	36	50.7	149	56.9	.354
	No	35	49.3	113	43.1	
	Total	71	100	262	100	
Symptoms severity	Mild	15	41.7	43	28.9	.201
	Moderate	16	44.4	68	45.6	
	Severe	5	13.9	38	25.5	
	Total	36	100	149	100	
Family loss due to COVID	Yes	12	16.9	42	16	.860
	No	59	83.1	220	84	
	Total	71	100	262	100	
Child's age	0−3	13	18.6	49	19.1	.275
	4−7	16	22.9	87	33.9	
	8−11	15	21.4	54	21	
	12−15	18	25.7	41	16	
	Above 15	8	11.4	26	10.1	
	Total	70	100	257	100	
The child has a chronic disease	Yes	3	4.2	9	3.4	.756
	No	68	95.8	252	96.6	
	Total	71	100	261	100	
The child received the Flu vaccine	Yes	23	32.4	70	26.8	.354
	No	48	67.6	191	73.2	
	Total	71	100	261	100	
If no	I have the intention to vaccinate my child	32	69.6	22	12	<.001
	I don't have the intention to vaccinate my child	14	30.4	162	88	
	Total	46	100	184	100	
Did your child receive all the prescribed vaccinations according to the national vaccination program	Yes	62	88.6	213	81.3	.211
	No	8	11.4	49	18.7	
	Total	70	100	262	100	

Abbreviation: COVID‐19, coronavirus infection 2019.

**Figure 2:**
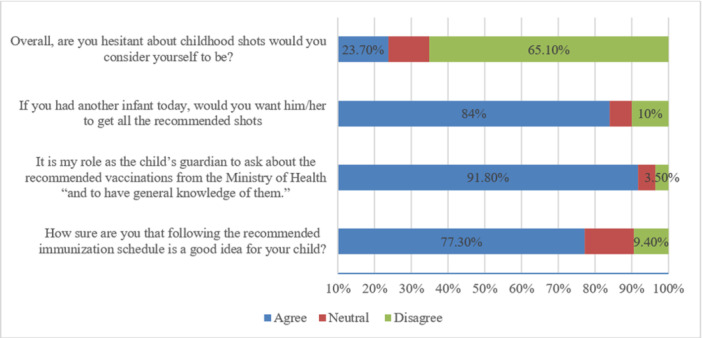
Attitudes toward vaccines in general.

Figure 2 demonstrates the participants' attitudes toward vaccines in general, the vast majority of the participants have a good attitude toward vaccines in general.

**Figure 3:**
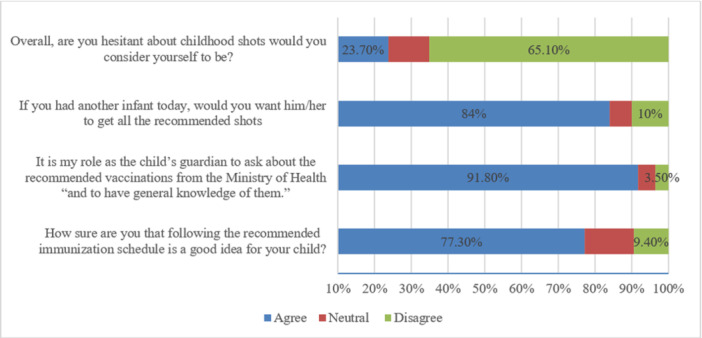
Beliefs about the safety and effectiveness of the COVID‐19 vaccine. COVID‐19, coronavirus infection 2019.

The beliefs about the safety and effectiveness of the COVID‐19 vaccine were illustrated in Figure 3. There are still some concerns regarding the COVID‐19 vaccine's safety for children based on parents' perspectives. Many parents believe that children should acquire immunity through illness rather than receiving the COVID‐19 vaccine. Additionally, they express concerns about potential serious side effects and the overall safety of the vaccine for children.

## 8 DISCUSSION

In the context of vaccine hesitation, this study provided important insights for understanding the willingness of parents to vaccinate their cancer patients' children against COVID‐19 and elucidating and identifying relevant factors to improve the vaccination rate for such children in Jordan.

Understanding factors associated with parents' intentions to vaccinate children during a pandemic may support public health officials' efforts toward broader acceptance of the vaccine and thus reach a higher level of immunity in the population. In addition to that, vaccination of children will help to boost their immunity, reduce the risk of disease, and enable them to participate in other activities—sports, games, socializing with friends—that are so important to their health and development.^7^ The uptake of a COVID‐19 vaccine among children will be crucial in limiting the spread of the disease as herd immunity may require vaccine coverage for up to 80% of the population.^30^

The results of the present study examining parents' acceptance of their children's COVID‐19 vaccination indicated that COVID‐19 immunization for children was not widely accepted. Parental hesitancy toward a future COVID‐19 vaccine is higher than their hesitancy toward children's seasonal flu vaccination. Meanwhile, recent data shows that 6.1% of US parents are hesitant about routine vaccines, and 25.8% of parents are hesitant about the annual influenza vaccine.^8^ Indeed, only a small fraction of the respondents had their child already vaccinated with COVID‐19 vaccination compared to the general population.

Our study found that mothers were more willing than fathers to vaccinate their children. This may be because mothers spend more time with their children than fathers and are more concerned about their children's health‐related illnesses.^31^ This finding can be applied to create strategies such as providing gender‐specific parental education campaigns to maximize the acceptability of vaccines, for example, conduct in‐depth research to understand the specific concerns, beliefs, and barriers related to vaccination among fathers in the target population, and develop educational materials and messaging that address the concerns and priorities of both mothers and fathers. For mothers, emphasize the importance of vaccination in protecting their children's health and well‐being, drawing on their nurturing instincts and desire to safeguard their children. For fathers, focus on the role of vaccination in preventing the spread of infectious diseases and maintaining family and community health.

In the present study, the results indicate that the majority of parents base their decision on vaccinating their children with the COVID‐19 vaccine on the advice provided by pediatricians. A smaller proportion of parents, rely on research findings and scientific references to inform their decision‐making process. Additionally, the opinions of older people and relatives, as well as information disseminated through media and social media channels, play a relatively minor role, considering them in their decision‐making process.

Concerns about vaccines' safety and effectiveness and the perception that children were at low risk of infection were also reasons for the tendency to reject our study. Concern about side effects was an important factor in parents. Negative attitudes toward vaccines were shown by participants due to their thoughts about developing symptoms in response to the vaccine, similar to those of the disease itself. It is possible to explain this result by negative or wrong information resulting from the media. Many people talked about vaccination from different perspectives, which confused the public.

In addition to identifying demographic groups that may be hesitant about a COVID‐19 vaccine, we also characterized the association between demographic factors and COVID‐19 vaccine hesitancy. Parents aged 45–54 years and children aged above 15 years old exhibited the highest vaccination rates. Additionally, we identified significant associations between children's vaccination status and parental chronic disease status, as well as parents receiving the COVID‐19 vaccine themselves. These findings suggest that demographic characteristics and personal health factors may play significant roles in shaping parental decisions regarding COVID‐19 vaccination for their children. Additionally, it emphasizes the need for tailored interventions, targeted health education efforts, community engagement strategies, and policy considerations to address vaccine hesitancy and promote COVID‐19 vaccination uptake among children, particularly within specific demographic groups identified in the study.

Thus, public health workers should focus on providing more education on improving other parents' overall confidence in the safety and effectiveness of the vaccine, as this is the main reason that leads to the rejection of the COVID‐19 vaccine, such as targeted messaging and educational campaigns that emphasize the importance of COVID‐19 vaccination for children, particularly among parents with chronic health conditions. These campaigns could highlight the protective benefits of vaccination for both children and their families, emphasizing the role of vaccination in reducing the risk of severe illness and complications associated with COVID‐19, especially among vulnerable populations. Additionally, healthcare providers can play a pivotal role in addressing parental concerns and promoting vaccine confidence by engaging in open and empathetic conversations with parents, addressing their specific concerns, and providing accurate and accessible information about the safety and efficacy of the COVID‐19 vaccine for children.

Similar factors were reported regarding the uptake of children's COVID‐19 vaccination by Canadian parents such as the age of the child, children who are up‐to‐date on their immunization schedule, and if the child or caregiver was vaccinated against influenza in the past year.^32^

Our study found no significant correlation between income, education level, and vaccination intention; this may be because the vaccine was given for free, and the COVID‐19 vaccine‐related information is widely disseminated such that people with different education levels have little difference in understanding it. Similar to a Canadian study, level of education was not a factor associated with intent to vaccinate against COVID‐19 in our study.^32^

In line with our results, a study from the United Kingdom with parents of children younger than 18 months found that they were more hesitant to have their child vaccinated than to get vaccinated themselves.^24^ A multinational study showed that parents were more willing to vaccinate their child when they were older, which is also aligned with our study.^32^

Our study participants were more likely to accept a COVID‐19 vaccine for themselves than their children, similar to the results reported by Bell et al.^24^

When reviewing the current literature on this topic, we found much higher vaccine acceptability results in several other studies performed in other countries at different times, including the United States, Germany, Australia, and China. In the United States, in a cross‐sectional online survey of parents with children <18 years old, overall, 33% of parents reported vaccine hesitancy for their child, the highest rates of hesitancy toward a future COVID‐19 vaccine were found in demographic groups that have been the most severely affected by the pandemic,^33^ while our results revealed that there is no association between parental decision and parental severity of COVID‐19 infection, it's plausible that parents base their vaccination decisions on factors other than their own experience with COVID‐19, additionally, the severity of parental COVID‐19 infection may not necessarily correlate with their perceived risk or urgency to vaccinate their children. Parents may perceive COVID‐19 vaccination for their children as a preventive measure to safeguard their health and mitigate the risk of transmission within their families and communities, irrespective of their own infection severity.

Our study found low parental intent for child vaccination, contrasting with a German study that investigated parents' intention to get vaccinated and to have their child vaccinated against COVID‐19, 51% intended to have their children vaccinated.^30^ Additionally, our findings diverged from Australian study which investigated the parents' vaccine intentions for themselves and their children and found that 48% intend to vaccinate their children.^34^ Furthermore, an online survey conducted in the United Kingdom showed that 55.8% of parents would accept COVID‐19 vaccination for their child aged 18 months or under.^25^ These discrepancies highlight variability in vaccine acceptance across regions, suggesting the need for tailored interventions to address vaccine hesitancy among parents globally.

Our findings starkly contrast with the results from studies conducted in China. For instance, a study from China by Zhang et al., showed that the prevalence of parents' acceptance who were factory workers of COVID‐19 vaccination for their children was 72.6%.^28^ Moreover, another Chinese study investigated parental acceptability of COVID‐19 vaccination for children under the age of 18 years among Chinese parents who are healthcare workers (HCWs), among the participants, 44.5% reported that they would likely or very likely to have their child under the age of 18 years take up COVID‐19 vaccination,^24^ indicating variability in vaccine acceptance across populations.

Our study's finding that only 18.6% of parents of children aged 3–6 intend to vaccinate their child with the flu vaccine contrasts sharply with a survey of Chinese parents of children aged 3–6 showed that 86.75% were willing to vaccinate their children with the COVID‐19 vaccine.^7^

In comparing our study's results to those of other relevant studies, it becomes evident that while there are similarities in some findings, there are also notable differences that highlight the nuanced nature of parental vaccine decision‐making, particularly regarding COVID‐19 vaccination for children. For instance, our study revealed significant associations between parental and child age, parental chronic disease status, parental COVID‐19 vaccination, and children's vaccination rates among those with cancer. These findings align with broader trends observed in vaccine acceptance behavior among parents. However, a study conducted in China found that factors such as maternal gender, perception of high‐risk status, active engagement with vaccine‐related information, belief in vaccine safety, and perceived efficacy were key drivers of parental willingness to vaccinate against COVID‐19.^7^

In contrast to our findings, a study conducted in Poland regarding pediatric vaccination, 44% of parents want to vaccinate their child as soon as possible, but expressed similar concerns about adverse effects and insufficient testing in pediatric populations.^29^

Many studies around the world have reported less hesitancy toward the COVID‐19 vaccine among parents toward children vaccination compared to what our study found. An online questionnaire evaluating parental hesitancy in vaccinating their child against COVID‐19 was conducted in China, 52.5% were hesitant, and mothers exhibited a greater proportion of vaccine hesitancy than fathers did^35^ in contrast to our study, which found that mothers were actually more willing than fathers to vaccinate their children.

It appears there is a discrepancy regarding parental vaccine hesitancy between parents of pediatric ≥12 years and parents of children <12 years. In a cross‐sectional study to investigate the knowledge, attitude, and intention to vaccinate children <18 years in Italian families, among families of pediatric ≥12 years, 41.2% would not vaccinate their child, while among families of children <12 years, 36.1% would not vaccinate,^23^ in contrast, our study found that parents of pediatric ≥12 years were actually less hesitant to vaccinate their children compared to parents of children <12 years. Additionally, their determinants of intention to vaccinate both age groups were the perceived safety and efficacy of vaccines and the perceived risk of transmitting the infection to adults.^23^

Comparing our study to others reveals consistent patterns of parental vaccine hesitancy toward COVID‐19 vaccination for children. In New York, around half of the participants were hesitant about vaccinating their children, lower income and less education were associated with greater parental vaccine hesitancy/resistance; safety and lack of need were primary reasons for vaccine hesitancy/resistance. While a study investigated vaccine acceptance of COVID‐19 vaccines among a national sample of vaccine‐hesitant parents in the United States indicated a general unwillingness among participants to vaccinate their child and themselves, they also found that more educated parents were more likely to plan to vaccinate themselves and their child.^26^ These findings underscore the complex nature of vaccine hesitancy and suggest the need for targeted public health interventions.

In our study, we found that the child's pediatrician played a significant role in encouraging parents to vaccinate their children against COVID‐19. This highlights the importance of healthcare providers in addressing parental concerns and promoting vaccine confidence through open and empathetic communication. Similarly, a study conducted among HCWs in southern Italy found that the COVID‐19 pandemic had an impact on the use of preventive measures among HCWs, with a positive change observed in adherence to face mask‐wearing and hand hygiene practices. The study also emphasized the importance of educational messages and the promotion of vaccination among HCWs to reduce vaccination gaps and prevent the spread of infection. These findings underscore the critical role of healthcare providers in promoting vaccination and adherence to preventive measures during public health crises like the COVID‐19 pandemic.^36^

Therefore, Jordanian parents' willingness to vaccinate their children against COVID‐19 may be different from parents from other countries, considering that COVID‐19 is effectively controlled in Jordan during the period of this study.

## 9 LIMITATIONS

First, the study relies on self‐reported data, introducing the possibility of recall bias and social desirability bias in participants' responses. Additionally, the cross‐sectional design limits the establishment of causation and only provides a snapshot of attitudes at a specific point in time. The study was conducted at a single cancer center in Jordan, potentially limiting the generalizability of the findings to a broader population. Furthermore, the study primarily focuses on parental perspectives, potentially overlooking the child's viewpoint. Despite these limitations, efforts were made to ensure a diverse sample, and the findings contribute valuable insights into parental attitudes toward COVID‐19 vaccination in the context of pediatric cancer.

## 10 CONCLUSION

A large proportion of parents are hesitant about the COVID‐19 vaccine because they are less confident in its effectiveness, safety, and whether it is essential for their child.

This finding emphasizes the importance of communication and education to address vaccination hesitancy. In essence, there is room for improvement of parents' knowledge about the role of children in sustaining the circulation of infection, the safety of vaccines, and, most importantly, the way vaccines are developed and tested in children, which is the main reason for a negative intention to vaccinate and for vaccine hesitancy in our sample. These areas of knowledge need to be better addressed to create a solid foundation for public trust in COVID‐19 vaccines.

## AUTHOR CONTRIBUTIONS

**Sawsan Mubarak**: Conceptualization; data curation; methodology; supervision; validation; visualization. **Hadeel AlGhawire**: Data curation; investigation; writing—original draft. **Sumaiah AlNaimat**: Formal analysis; investigation; visualization; writing—review and editing.

## CONFLICT OF INTEREST STATEMENT

The authors declare no conflict of interest.

## ETHICS STATEMENT

This study was approved by the Institutional Review Board Committee of King Hussein Cancer Center (no. 21 KHCC 180).

## Data Availability

The data that supports the findings of this study are available on request from the corresponding author.
